# Early intubation and patient-centered outcomes in septic shock

**DOI:** 10.1186/s13054-022-04152-4

**Published:** 2022-10-03

**Authors:** Jianmin Qu, Yanfei Shen, Huijuan Zhang

**Affiliations:** 1 Department of Intensive Care, Tongxiang First People’s Hospital, Jiaochang Road 1918, 314500, Tongxiang, Zhejiang P.R. China; 2 Department of Intensive Care, Zhejiang Hospital, Gudun Road 1229, 310013 Hangzhou, Zhejiang P.R. China

Dear Editor,

In a recent study [1], Dr. Mellado-Artigas investigated the efficacy of early intubation (within eight hours after vasopressor use) in patients with septic shock. After propensity score matching (PSM), they reported that compared to non‑early intubation, early intubation did not improve in-hospital mortality or ICU/hospital length of stay in the matched cohort. We noted that in the non-early intubation group, only 33% (27/78) of these patients finally needed intubation. Therefore, the comparison between early intubation and non-early intubation group is actually the comparison between patients with early intubation and those (67%) who do not need intubation mixed with a small proportion (33%) of patients with non-early intubation. In clinical practice, patients who do not need intubation always tend to have milder conditions and a relatively better prognosis than those who need intubation. Therefore, we speculated that the non-significant comparisons in the current study may have been affected by several factors.

First, the major issue with PSM in the current study is the external validity. A total of 137 patients were included in the early intubation group, 43% (59/137) of whom were excluded from PSM. In Table 1, we note that in the early intubation group, the mortality was significantly higher in patients excluded from PSM than that in those kept in the PSM (41/59 (69%) vs. 35/78 (45%), *p* = 0.004). On the contrary, in the non-early intubation group, the mortality was significantly lower in patients excluded from PSM than that in those kept in the PSM (85/520 (16.3%) vs. 26/78 (33%), *p* < 0.001). This means that only mild patients in the early intubation group and severe patients in the non-early intubation group were included in the PSM analysis. Therefore, the exclusion of these patients in PSM may lead to poor sample representation and affect the external validity of these results.

**Table 1:** Mortality comparisons between patients kept and excluded from the PSM in two groups

	Used for PSM	Excluded from PSM	*p*
Early intubation group	N = 78	N = 59	
Mortality rate* [n (%)]	35 (45.8%)	41 (69.0%)	0.004
Non-early intubation group	N = 78	N = 520	
Mortality rate [n (%)]	26 (33.3%)	85 (16.3%)	< 0.001
P^#^	0.140	< 0.001	

*The overall mortality rate was extracted from their supplementary Table S2 (mortality by day 60)

^#^Comparisons between early intubation and non-early intubation groups

Second, a total of 22 variables were included in the PSM analysis. Although most of these selected variables were balanced in the matched cohort, hidden bias [2–4] due to unmeasured confounders likely remains. For instance, severe hypoxemia was one common indication for intubation in clinical practice. Aiming to identify patients with similar oxygenation status, the worst PaO_2_/FiO_2_ level should be used for matching in the PSM. However, in the current matched cohort, the mean PaO_2_/FiO_2_ was 141 and 153 mmHg in the early intubation and non-early intubation groups, which unlikely reflects the worst oxygenation status. Therefore, some clinical or laboratory indications for intubation may be missing in the PSM analysis. In addition, the balance of several binary variables should not be measured using standardized mean differences (SMD). For instance, although the SMD is small, the accessory muscle use (43/78 (0.55%) vs. 36/78 (0.46%)) is still not well balanced. Sensitivity analysis for unmeasured confounding, such as Rosenbaum bounds (Gamma value) [5] or other models [2], should be reported to quantify the hidden bias.

## Data Availability

Not applicable.
