# Supplementary material for: Early intubation and patient-centered outcomes in septic shock
Source: Crit Care. 2022 Oct 3;26:299. doi: 10.1186/s13054-022-04152-4 (PMC9528133; doi:10.1186/s13054-022-04152-4)
Supplement: Related texts

# Authors reply to: Early intubation and patient-centered outcomes in septic shock

**Authors:** Ricard Mellado-Artigas, Carlos Ferrando, Frédéric Martino, Agathe Delbove, Bruno L. Ferreyro, Cedric Darreau, Sophie Jacquier, Laurent Brochard, Nicolas Lerolle

Dear Editor,

We thank Dr. Hu and colleagues for their interest in our work and for the time taken to describe some concerns around our results [1].

In first place, the authors mentioned that our analysis compared not only intubated patients but also a subgroup of patients who never received mechanical ventilation. This approach, also known as the target trial framework, was followed to aim at resembling the construction of a hypothetical trial [6]; where patients would have been randomized to two different strategies, namely one including expeditious intubation and another one focusing on a more conservative approach. In the latter some patients would have likely ended up receiving intubation while many others would have not. In our opinion and in others’, only including patients receiving intubation might result in biased estimates [7].

Second, the authors pointed out that our PSM might have negatively affected the external validity of our results since only a subgroup of patients was included in the matched analysis. We fully agree with the authors, but we cannot help but remind that we repeatedly warranted caution about the risk of extrapolating the results to the whole population, which would be similar to negate the usefulness of mechanical ventilation in critical illness. In our analysis, we were only able to include 78 out of 137 (57%) of those intubated in the first 8 h and the findings only apply to this subgroup. Although, it would have been interesting to estimate the effect of early intubation in the whole population, it was simply not possible since the higher disease severity on the other half of the early-intubated subgroup did not allow for a proper matching.

Third, the authors raised the concern that balance might have not been achieved for several variables. Although the discussion about the number of variables and parameters to assess balance could be of great interest, space constraints limit it here. Nonetheless, for the present work, we developed several sensitivity analyses using overlap weighting, an inverse-probability of treatment weighting-based technique, which virtually eliminates imbalance between groups [8]. These analyses, although focused on a smaller subgroup also confirmed the previous findings.

Finally, we have computed the E-value which represents the magnitude of a potential unmeasured factor in the risk ratio scale to explain away the observed effect [9]. For the main hazard ratio (HR) to be 1, E-value was 1.92; for HR 0.90, E-value was 2.11 and for HR 0.80, E-value was 2.37.
